# Characterization of hsa_circ_0004277 as a New Biomarker for Acute Myeloid Leukemia via Circular RNA Profile and Bioinformatics Analysis

**DOI:** 10.3390/ijms18030597

**Published:** 2017-03-09

**Authors:** Wei Li, Chaoqin Zhong, Jun Jiao, Peng Li, Baoxia Cui, Chunyan Ji, Daoxin Ma

**Affiliations:** 1 Department of Hematology, Qilu Hospital, Shandong University, Jinan 250012, China; liwei_medicine@163.com (W.L.); zhongchq07@126.com (C.Z.); pengli85@163.com (P.L.); jichunyan@sdu.edu.cn (C.J.); 2 Department of Obstetrics and Gynecology, Qilu Hospital, Shandong University, Jinan 250012, China; jiaojun206@163.com (J.J.); cuibaoxia@sdu.edu.cn (B.C.)

**Keywords:** circRNAs, AML, hsa_circ_0004277, biomarker

## Abstract

Circular RNAs (circRNAs) represent a widespread class of non-coding RNAs, which drew little attention in the past. Recently, limited data showed their promising future to act as biomarkers in human cancer, but the characteristics and functions remain largely unknown in hematopoietic malignancies, especially in leukemia. In this study, with the help of circRNA microarray, we demonstrated the expression profile of circRNAs in acute myeloid leukemia (AML) patients, and identified a large number of circRNAs possibly expressed in a leukemia specific manner. We also described a circRNA signature related to AML risk-status based on the bioinformatics prediction. In particular, a downregulated circRNA, hsa_circ_0004277, was characterized and functionally evaluated in a cohort of 115 human samples, thus offering a potential diagnostic marker and treatment target in AML. Interestingly, we found chemotherapy could significantly restore the expression of hsa_circ_0004277, indicating the increasing level of hsa_circ_0004277 was associated with successful treatment. Furthermore, a detailed circRNA–miRNA–mRNA interaction network was presented for hsa_circ_0004277, allowing us to better understand its underlying mechanisms for function in AML.

## 1. Introduction

Acute myeloid leukemia (AML) represents a group of myeloid malignancies characterized by acquired genetic abnormalities in hematopoietic progenitors. It shows remarkably complex biological and clinical heterogeneity in patients. Advances in molecular genetics promote the recognition of AML prognostic biomarkers, among which cytogenetic results and molecular abnormalities are considered as the most important factors. Nearly half of AML patients are found without cytogenetic abnormality, and constitutes the main part of intermediate-risk AML category [1]. However, these cytogenetically normal AML (CN-AML) patients can be divided into different subgroups based on molecular markers such as FLT3-ITD and NPM1 mutation. Patients with FLT3-ITD mutation are in the poor-risk group, whereas patients with NPM1 mutation in the absence of FLT3-ITD or isolated biallelic CEBPA mutation are in the better-risk group [2]. Currently, extensive research studies have been carried out to identify more prognostic biomarkers, focusing on various kinds of molecular signals: DNA mutations, aberrant expressions of microRNA, and long noncoding RNA (lncRNA) [3]. Hopefully, these potential biomarkers could be combined to significantly improve the diagnosis, prognosis, or monitoring of AML.

Circular RNAs (circRNAs), a big class of noncoding RNAs, were discovered from a few of transcribed genes over twenty years ago [4] and were initially considered as by-products of mis-spliced RNAs or splicing errors [5,6]. However, recent high-throughput sequencing and novel computational approaches demonstrated that they are more likely to be widespread products of regulated back-spliced RNAs with distinct sets of *cis*-elements and/or trans-factors [7]. circRNAs have a closed continuous loops configuration due to a non-random back-splice event that involves a covalent junction between the 3′ and 5′ ends [8]. This structure is typically non-polyadenylated, with a resistance to exonucleases that makes them more stable compared with their linear counterparts. Nowadays, circRNAs are known to be highly prevalent in the eukaryotic transcriptome, abundant, conserved, and stable in cytoplasm, and even in blood [9]. The expression of circRNA was found to be tissue/developmental-stage-specific [10,11] and also shown to act as miRNA sponges that regulate gene expression [8,12,13]. Furthermore, limited evidence suggested that circRNAs are associated with cancer and may play a significant part in its pathogenesis and diagnosis [14,15,16]. However, no data was reported regarding the roles of circRNAs in the development of AML or its response to treatment.

To demonstrate the expression profile of circRNAs in AML, we performed a circRNA microarray in AML patients and healthy controls for the first time, and presented an AML-specific circRNA feature, as well as a unique signature related to AML risk-status. In particular, we identified one novel circRNA, hsa_circ_0004277, as a potential diagnostic marker and treatment target in AML. Moreover, with the help of bioinformatics analysis, a detailed circRNA-miRNA-mRNA interaction network was described for hsa_circ_0004277, allowing us to better understand its underlying mechanisms for function in AML.

## 2. Results

### 2.1. Profile of circRNA Expression in AML Patients

**Figure 1:**
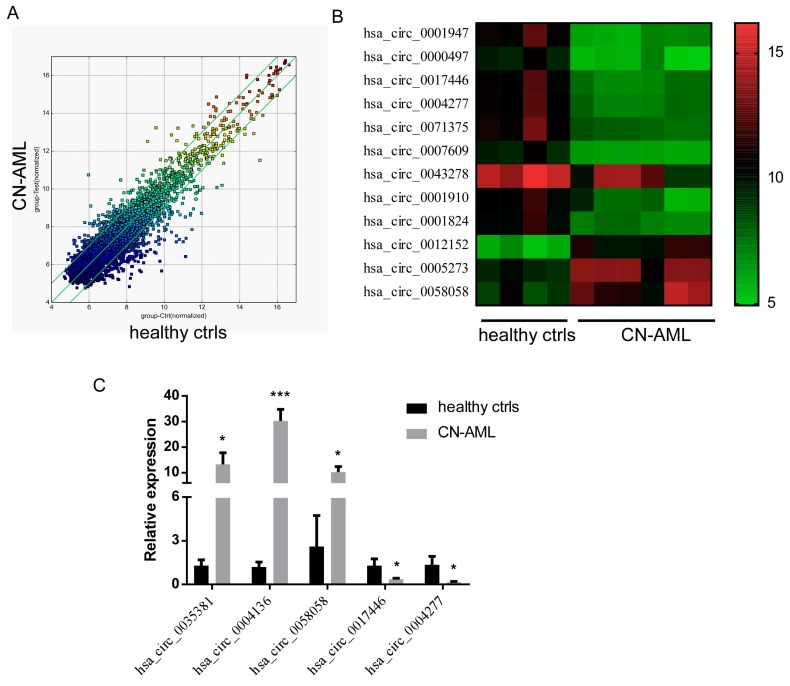
Circular RNA (circRNA) profile in acute myeloid leukemia (AML) and quantitative Real-Time PCR (qRT-PCR) validation. (**A**) Scatter plot showing the expression profile of 4573 circRNAs between the two compared groups. circRNAs above and below the border green line were expressed more than two-fold changes in AML patients than the healthy controls; (**B**) Clustered heatmap for 12 dysregulated circRNAs 10-fold more prevalent than the average of healthy controls; (**C**) Quantitative RT-PCR validation for five circRNAs from the microarray data. * *p* < 0.05, *** *p* < 0.001.

Firstly, we characterized circRNA transcripts using a microarray platform in 10 human samples, four healthy controls vs. six newly diagnosed CN-AML patients without prior treatment. The detailed clinical information of patients is available in Table 1. According to our intensity filtering system, 4573 out of 13,617 circRNAs were beyond detection and selected for further analysis. Among those 4573 circRNAs, 464 distinct circRNAs were found differentially expressed, in which 147 circRNAs were upregulated and 317 circRNAs were downregulated more than two-fold in CN-AML patients compared to healthy controls (Figure 1A). Furthermore, 12 dysregulated circRNAs were 10-fold more prevalent than the average of healthy controls (Figure 1B).

### 2.2. Validation of Differentially Expressed circRNAs from the Microarray Profile

We further confirmed the microarray results by quantitative Real-Time PCR (qRT-PCR) using the same cohort of patient and control samples as in microarrays. Five abundant circRNAs (hsa_circ_0035381, hsa_circ_0004136, hsa_circ_0058058, hsa_circ_0017446, hsa_circ_0004277) were chosen based on the significant difference and raw signal intensity of expression. As shown in Figure 1C, qRT-PCR results were consistent with the microarray results, as three circRNAs were upregulated and two were downregulated in AML patients, thus demonstrating high reliability of this circRNA profile.

### 2.3. Identification of circRNA Signature between Better-Risk and Poor-Risk AML Patients

As shown in Table 1, in the six CN-AML patients from the microarray, three were diagnosed as better-risk AML, while the other three were diagnosed as poor-risk AML, based on whether they had FLT3-ITD and NPM1 mutation or not. Among the 464 differentially expressed circRNAs we had found above, we kept on searching circRNAs that had significant difference between better-risk and poor-risk subgroups. The patterns we were interested in were quite clear, as long as candidate circRNAs showed progressive upregulation or downregulation in three groups in the following order: healthy controls, better-risk AML subgroup, and poor-risk subgroup. Obviously, “upregulation” or “downregulation” of candidate circRNAs should also reach statistical significance to meet the inclusive criteria (fold change cut-off: 2.0 and *p*-value cut-off: 0.05). By following those patterns, two upregulated circRNAs (hsa_circ_0035381, hsa_circ_0049657) and three downregulated circRNAs (hsa_circ_0001187, hsa_circ_0008078, hsa_circ_0001947) were found within the whole profile (Table 2). This result showed us a circRNA signature not only presents in AML, but also potentially contributes to AML risk-status evaluation.

### 2.4. Predicted Characteristics of the circRNA Signature Related to AML Risk-Status

**Figure 2:**
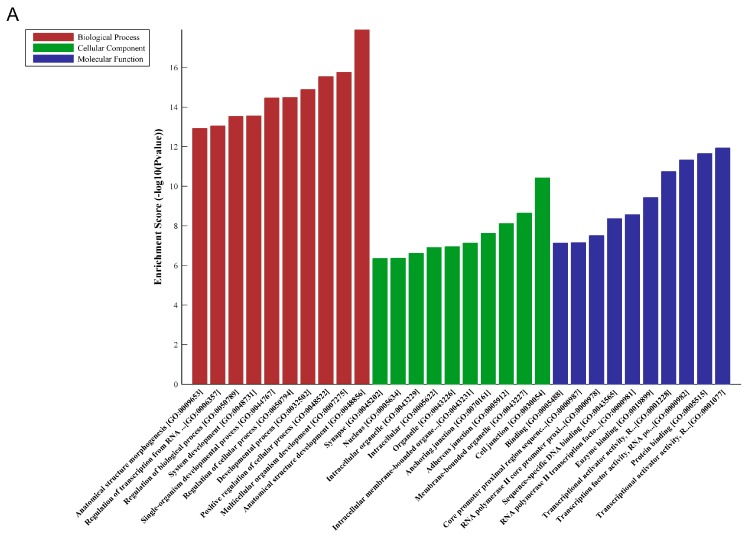

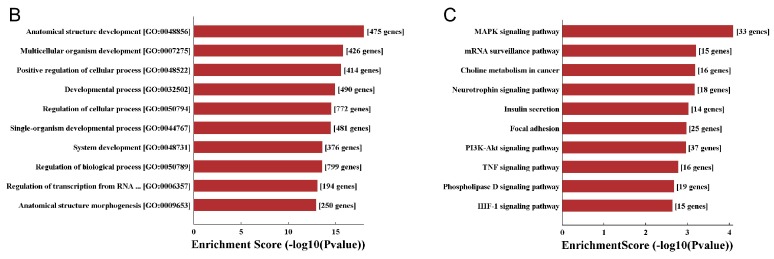
Gene Oncology (GO) and Kyoto Encyclopedia of Genes and Genomes (KEGG) pathway analysis in the circRNA signature related to AML risk-status; (**A**,**B**) GO enrichment corresponds to the five altered circRNAs related to AML risk-status; (**C**) KEGG pathway analysis corresponds to the five altered circRNAs related to AML risk-status.

As reported, one of the well-known regulatory functions of circRNA is to serve as miRNA sponges and competitively suppress its activity. For this purpose, we identified and ranked miRNAs for those five circRNAs related to AML risk-status. The five highest-ranking candidate miRNAs for each circRNA were included through specific base pairing. Furthermore, Gene Oncology (GO) and Kyoto Encyclopedia of Genes and Genomes (KEGG) pathway analysis was performed to investigate the functions of their target genes. The results revealed that the target genes related to this circRNA signature participated in various biological processes, such as developmental process, regulation of cellular process and cell junction (Figure 2A,B). Some vital pathways could also be influenced by the altered circRNAs, including MAP kinase (MAPK) signaling pathway, Phosphatidylinositol 3-Kinase–AKT (PI3K-Akt) signaling pathway, and Hypoxia-inducible factor 1 (HIF-1) signaling pathway (Figure 2C).

### 2.5. Identification of Novel hsa_circ_0004277 in AML

**Figure 3:**
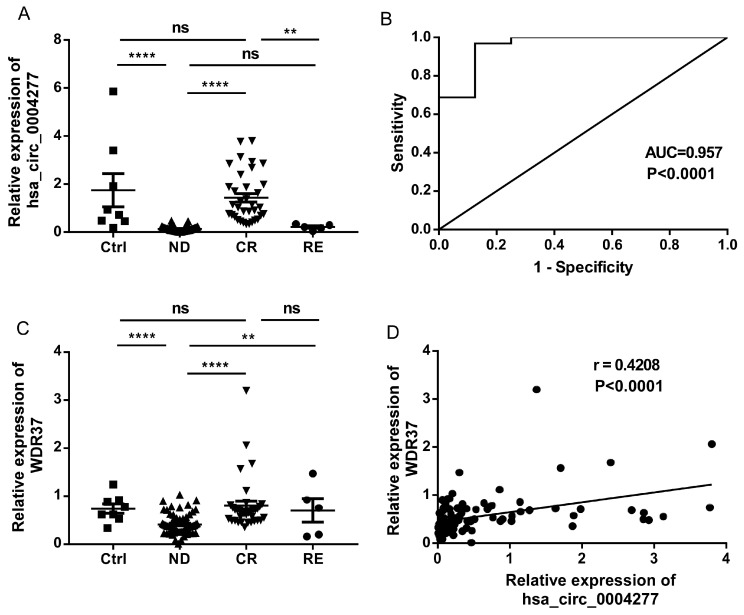

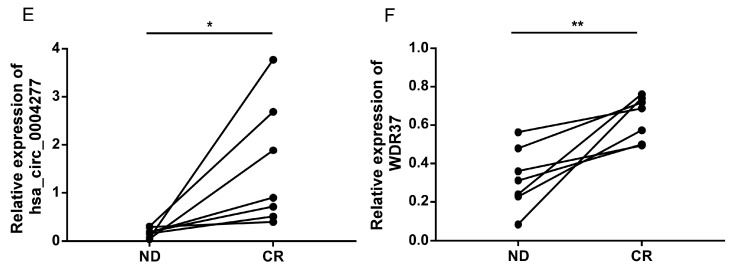
Expression of hsa_circ_0004277 and WDR37 in AML. (**A**) hsa_circ_0004277 expression measured by qRT-PCR in eight healthy controls and 107 AML patients at different stages (ND, *n* = 67; CR, *n* = 35; RE, *n* = 5); (**B**) receiver-operating characteristic (ROC) analysis for hsa_circ_0004277 in 67 ND AML patients; (**C**) WDR37 expression measured by qRT-PCR in eight healthy controls and 107 AML patients at different stages (ND, *n* = 67; CR, *n* = 35; RE, *n* = 5); (**D**) correlation between hsa_circ_0004277 and WDR37 in 107 AML patients at different stages; (**E**,**F**) hsa_circ_0004277 and WDR37 expressions were measured in matched-pair samples acquired from seven available follow-up AML patients at the time when they were at ND and CR stage. ND, newly diagnosed; CR, complete remission; RE, relapsed-refractory; ns, not significant. * *p* < 0.05, ** *p* < 0.01, **** *p* < 0.0001.

During our validation of the microarray results, we noted one of the most significantly downregulated circRNAs in AML, hsa_circ_0004277, has a particularly high expression in human samples, which could be an advantage for further exploration [17]. Therefore, hsa_circ_0004277 was selected for our large sample validation. The expression of hsa_circ_0004277 was then determined by qRT-PCR in eight healthy controls and 107 AML patients at different stages. A much lower expression level of hsa_circ_0004277 was found in newly diagnosed AML patients without prior treatment (ND group) compared to the healthy controls (ctrl group) (Figure 3A, *p* < 0.0001). Meanwhile, patients achieved complete remission (CR group) after treatment restored the expression of hsa_circ_0004277, showing no difference compared to the control group. However, in the relapsed-refractory patients (RE group) after CR stage, downregulated hsa_circ_0004277 expression was observed again (Figure 3A). These results showed us a dynamic expression of hsa_circ_0004277 according to the progressive stage of AML, thus providing us a potential diagnostic biomarker in AML.

Next, we performed the receiver-operating characteristic (ROC) curve analysis to examine its potential diagnostic value in distinguishing individuals with AML from healthy individuals. Area under the ROC curve (AUC) was 0.957 (Figure 3B), indicating hsa_circ_0004277 had a promising potential as a biomarker. Associations between hsa_circ_0004277 and several AML clinical features are shown in Table 3, demonstrating no significant differences in the age, gender, French-American-British (FAB) classification, or karyotype subtypes, which implies that hsa_circ_0004277 might function in an independent way.

As reported, relationships between circular and linear RNA isoforms were always complicated and worthwhile to explore [18,19]. The linear isoform of hsa_circ_0004277 is *WDR37*, located at chr10:1125950-1126416. Little is known about this gene in general, and barely anything in specific regards to AML; therefore, we started with checking its expression in AML in the same cohort of samples as in Figure 3A. Similar results like hsa_circ_000427 were found about WDR37 in AML, with lower expression in the ND group, and higher expressions in the control and CR groups (Figure 3C). Unfortunately, WDR37 expression in the RE group (*n* = 5) had a wide range, not valid enough to show the differences with other groups. Furthermore, a positive correlation between hsa_circ_0004277 and WDR37 was observed in all the AML patients at different stages (Figure 3D), which was consistent with the circRNA report concerning esophageal squamous cell carcinoma [19].

### 2.6. Effects of Chemotherapy on hsa_circ_0004277 Expression in AML Patients

To further understand the influence of chemotherapy on circRNAs, we followed the whole treatment process of seven AML patients undergoing successful standard induction chemotherapy. In matched-pair bone marrow (BM) samples acquired from those seven AML patients at both ND and CR stage, we demonstrated a significant increase of hsa_circ_0004277 expression in CR stage compared with ND stage (Figure 3E), suggesting the restoration of dysregulated hsa_circ_0004277 expressions after chemotherapy. At the same time, a significant increase of WDR37 expression was also found in patients’ CR stage compared with their ND stage (Figure 3F).

### 2.7. MicroRNA Prediction and Downstream Bioinformatics Analysis for hsa_circ_0004277

**Figure 4:**
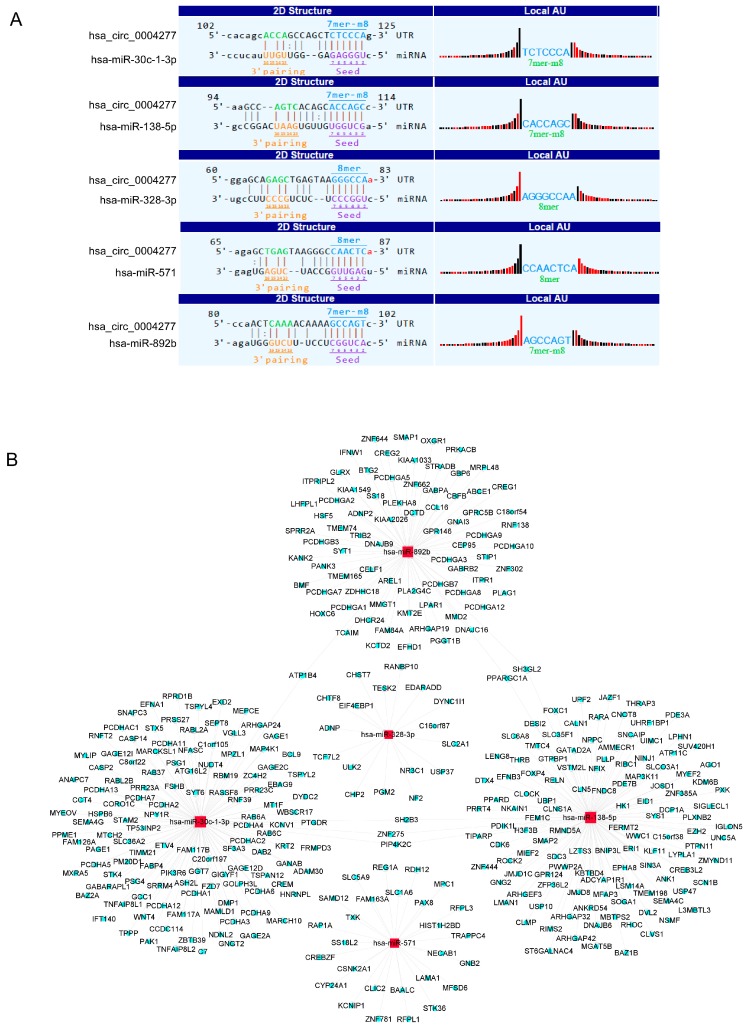
Bioinformatics prediction of hsa_circ_0004277 in AML. (**A**) The five highest-ranking candidate miRNAs matched hsa_circ_0004277; (**B**) circRNA-miRNA-mRNA network of hsa_circ_0004277, focused on the five highest-ranking candidate miRNAs.

Considering what we have seen above, hsa_circ_0004277 is very likely to be a special AML-related circRNA. We assumed that hsa_circ_0004277 could also act as a miRNA sponge and regulate its own circRNA-miRNA-mRNA network. The molecular interactions between hsa_circ_0004277 and its top five miRNA targets are displayed in Figure 4A. circRNA-miRNA-mRNA/gene network showed that hsa-miR-138-5p and hsa-miR-30c-1-3p exhibited the most complicated interactions (Figure 4B), followed by hsa-miR-892b, hsa-miR-571, and hsa-miR-328-3p. Among hundreds of mRNAs/genes identified from sequence-pairing with those five miRNAs, six of them were targeted by more than one miRNA: SH3GL2, *PPARGC1A*, *PIP4K2C*, *SH2B3*, *ZNF275*, and *ATP1B4*, which could be our main focus for further study.

## 3. Discussion

Recently, circRNAs have been a hotspot in the field of RNA, although the understanding of its nature and extent involved in biological events is still very limited. The formation of circRNAs is comparatively clearer than other aspects. To date, two mechanisms are proposed to form mammalian exonic circRNA: lariat-driven circularization and intron-pairing-driven circularization [20,21,22]. The main difference would be the first one featured by forming a lariat with restricted structure, while the second one refers to the pairing of a downstream splice donor with an upstream splice acceptor. Then, back-splicing is involved in both mechanisms by the canonical spliceosome. At last, this covalently closed loop structures are formed, with neither 5′ to 3′ polarity nor a polyadenylated tail.

It is reported that circRNAs are widely expressed in human cells, and their expression can be higher by 10-fold or more than their linear counterparts [22]. Due to the resistance to exonuclease, circRNAs are more stable and can be enriched by exonuclease exposure. Therefore, compared with other noncoding RNAs such as miRNAs and lncRNAs, the unique structure, high stability, and specific expression patterns provide circRNAs with the characteristics to be the ideal prognostic biomarkers and potential therapy targets. In this study, we firstly demonstrated the expression profile of circRNAs in AML patients, and identified a large number of circular RNAs possibly expressed in a leukemia specific manner, among which 12 circRNAs are 10-fold more prevalent than the average of healthy controls.

As to circRNA function, one of the most novel discoveries would be circRNAs acting as miRNA sponges. Certain circRNAs can bind and negatively influence miRNAs, which are substantially involved in the competing endogenous RNA (ceRNA) network, thereby regulating linear RNA transcription and protein production. Thomas et al. reported that circRNA ciRS-7 could strongly suppress miR-7 activity, resulting in increased levels of miR-7 targets [8,12]. Another identified miRNA sponge was sex-determining region Y (SRY), which could interact with miR-138 by 16 putative target sites [12]. Other functional possibilities include regulating gene expression at the transcriptional or post-transcriptional level [23], as well as encoding proteins with functions distinct from those of their canonical counterparts [24,25].

Besides profiling circRNAs between AML patients and healthy controls, our microarray designs also offer comparisons between better-risk and poor-risk AML subgroups. Five circRNAs were found to be dysregulated especially in the poor-risk subgroup, which can potentially contribute to AML risk-status evaluation. Combined with the sponge theory above, we further predicted their potential target miRNAs and performed GO and KEGG pathway analysis. The result revealed that these circRNAs contribute to an aggressive leukemia phenotype through certain biological mechanisms and pathways controlled by MAPK, PI3K-Akt, and so on. In addition to the most popular “sponge” theory, other possible mechanisms for those five circRNAs linked with AML risk-status remain comparatively unclear. The complicated interaction between the circular and the linear isoforms could be a possibility. It is reported one circRNA, cirZKSCAN1, varied in hepatocellular carcinoma patients with different tumor numbers, cirrhosis, vascular invasion, or the tumor grade, and furthermore, ZKSCAN1 mRNA and circZKSCAN1 cooperated closely with one another [26]. It became more interesting when we found NFIX, the linear isoform of hsa_circ_0049657 from Table 2, could modulate the differentiation of B cells and myeloid cells, which was associated with transcriptional changes in a number of myeloid and lymphoid lineage specific genes [27]. Therefore, the functions of *NFIX* and hsa_circ_0049657 could be worth exploring in our future research projects.

Based on the findings of Denzler et al., low levels of circRNAs may not be sufficient to affect the target miRNAs [17]. Therefore, one of the most significantly downregulated and also abundant circRNAs in the AML group from the microarray result, hsa_circ_0004277, was selected for further investigation. We next validated the aberrant expression of this circRNA using a large cohort of human BM samples, and a much lower expression level of hsa_circ_0004277 was found in the AML ND group than the ctrl group, in accordance with initial microarray results. By checking the expression of hsa_circ_0004277 in AML subgroups, we further compared the ND group with the CR group. It is promising to see that patients who achieved CR after treatment restored the expression of hsa_circ_0004277 showed no significant difference to the ctrl group. This result was verified in matched-pair BM samples acquired from seven AML patients at both ND and CR stage, which also presented us with results indicating the influence of chemotherapy on hsa_circ_0004277 expression. These results clearly showed a dynamic expression of hsa_circ_0004277 according to the progressive stage of AML. Combined with the ROC curve analysis, hsa_circ_0004277 is very likely to be a diagnostic biomarker or therapeutic target in AML. Furthermore, the circRNA-miRNA-mRNA interaction network of hsa_circ_0004277 from cytoscape analysis offered us several downstream gene-candidates, among which *SH3GL2* and *PPARGC1A* were reported to be involved in several solid tumors, and SH2B3 especially as “a new leukemia predisposition gene” [28]. These evidences indicate a functional relationship between hsa_circ_0004277 and gene expression in AML, suggesting to us that the downstream mechanisms deserve further investigation.

## 4. Materials and Methods

### 4.1. Human Samples and Cell Preparation

BM samples from 113 AML patients and 12 healthy controls were obtained following informed consent at Qilu Hospital, Shandong University. The study was conducted in accordance with the Declaration of Helsinki, and the protocol was approved by the Ethics Committee of Qilu Hospital (KYLL-2014-119; 25 February 2015). Ten samples were used for the circRNA microarray, and 115 samples were used for further investigation. Mononuclear cells from BM aspirates were separated by Ficoll-Paque Plus (Pharmacia LKB Biotechnology, Piscataway, NY, USA) density gradient centrifugation. Two million cells from each sample were collected for further procedure.

### 4.2. RNA Isolation, Labeling, and Hybridization

Total RNA was isolated from 10 samples using TRIzol reagent (Invitrogen, Carlsbad, CA, USA) according to the manufacturer’s protocol, and then treated with RNase R (Epicentre, Inc., Madison, WI, USA) to remove linear RNAs and enrich circRNAs. The enriched circRNAs were amplified and transcribed into fluorescent complementary RNA (cRNA) utilizing a random priming method (Arraystar Super RNA Labeling Kit; Arraystar, Rockville, MD, USA). Purification of the labeled RNAs was done by RNeasy Mini Kit (Qiagen, Hilden, Germany), and next the concentration and specific activity of the labeled cRNAs (pmol Cy3/μg cRNA) were measured by NanoDrop ND-1000. Five microliter 10× Blocking Agent and 1 μL of 25× Fragmentation Buffer were used to fragment 1 μg of each labeled cRNA, followed by heat at 60 °C for 30 min. Finally, 25 μL 2× Hybridization buffer was added to dilute the labeled cRNA and 50 μL of hybridization solution was dispensed into the gasket slide and assembled to the circRNA expression microarray slide, incubated for 17 h at 65 °C in an Agilent Hybridization Oven (Agilent Technologies, Santa Clara, CA, USA).

### 4.3. Microarray and Quality Control

After incubation, the hybridized arrays were washed, fixed, and scanned using the Agilent Scanner G2505C. Scanned images were imported into Agilent Feature Extraction software (version 11.0.1.1, Agilent Technologies) for raw data extraction. Quantile normalization and subsequent data processing were performed using the R software package. Next, low intensity filtering was performed and circRNAs with at least 4 out of 10 samples flagged in “P” or “M” (“all targets value”) were kept for further analysis. The Gene Expression Omnibus (GEO) accession number is GSE94591.

### 4.4. Real-Time Quantitative PCR Validation

Total RNA isolated from 115 samples was reversely transcribed into cDNA using MLV RTase cDNA Synthesis Kit (Takara, Japan). Quantitative RT-PCR was conducted in Roche Applied Science LightCycler 480II Real-time PCR systems (Roche Applied Science, Indianapolis, IN, USA) in accordance to the manufacturer’s instructions. A comparative cycle threshold (*C*_t_) method was used to analyze the gene expression level. Glyceraldehyde 3-phosphate dehydrogenase (GAPDH) was used as an internal control. Primer sequences were presented in Table 4.

### 4.5. MicroRNA Prediction and Functional Analysis

The circRNA-miRNA interaction was predicted with Arraystar’s home-made miRNA target prediction software based on TargetScan and miRanda. Cytoscape (available on: http: //www.cytoscape.org/) was applied to build a circRNA–miRNA–mRNA/gene interaction network. The predicted gene functions in the networks were annotated using Gene Oncology (GO) and Kyoto Encyclopedia of Genes and Genomes (KEGG) pathway analysis.

### 4.6. Statistics

Significant differences between different groups from the microarray were estimated by *t* test. circRNAs having fold changes ≥2 and *p*-values ≤ 0.05 were selected as the significantly differentially expressed ones. PCR data were presented as mean ± standard error of the mean (SEM). Significant differences were determined by Student *t* test and Spearman correlation test.

## 5. Conclusions

In conclusion, our study provided a unique circRNA profile in AML patients, based on which a substantial circRNA signature was demonstrated to indicate possible involvement in AML risk-status. Furthermore, we characterized and functionally evaluated one abundant circRNA, hsa_circ_0004277, thus offering a potential diagnostic marker and treatment target in AML.

## Figures and Tables

**Table 1:** Clinical information of human samples used in microarray.

Samples	Gender	Age	Diagnosis of FAB Subtypes	FLT-ITD3	NPM1	Karyotype	Risk Status
1	M	55	M5	+	+	Normal	Poor-risk
2	F	41	M5	+	−	Normal	Poor-risk
3	F	48	M4	+	−	Normal	Poor-risk
4	M	27	M5	−	+	Normal	Better-risk
5	F	58	M5	−	−	Normal	Better-risk
6	M	17	M4	−	+	Normal	Better-risk
7	M	35	Healthy control	N/A	N/A	N/A	N/A
8	M	47	Healthy control	N/A	N/A	N/A	N/A
9	F	52	Healthy control	N/A	N/A	N/A	N/A
10	F	28	Healthy control	N/A	N/A	N/A	N/A

M, male; F, female; FAB, French-American-British classification; FLT-ITD3, FMS-like tyrosine kinase-3; NPM1, Nucleophosmin 1; N/A, not applicable.

**Table 2:** Information of circRNAs related to AML risk-status.

circRNA	Regulation in AML	*p*-Value	Fold Change	circRNA Type	Chrom	Best Transcript	Gene Symbol
hsa_circ_0035381	up	0.006	3.661	exonic	chr15	uc002act.3	*PIGB*
hsa_circ_0049657	up	0.000	3.987	exonic	chr19	uc002mwg.2	*NFIX*
hsa_circ_0001187	down	0.000	6.465	exonic	chr21	uc011aeb.2	*DOPEY2*
hsa_circ_0008078	down	0.000	5.084	exonic	chr21	uc011aeb.2	*DOPEY2*
hsa_circ_0001947	down	0.000	25.993	exonic	chrX	uc004fco.3	*AFF2*

**Table 3:** Expression of hsa_circ_0004277 in different subgroups of newly diagnosed (ND) AML patients.

Features	No.	hsa_circ_0004277
**Age (years)**		
<43	32	0.14 ± 0.02
≥43	35	0.14 ± 0.02
*p*-Value	-	0.4756
**Gender**		
Male	26	0.12 ± 0.02
Female	41	0.15 ± 0.02
*p*-Value	-	0.0996
**FAB subtypes**		
M1	3	0.06 ± 0.01
M2	8	0.18 ± 0.05
M3	17	0.19 ± 0.10
M4	10	0.08 ± 0.02
M5	29	0.20 ± 0.07
*p*-Value	-	0.9021
**Karyotype**		
Better-risk	7	0.10 ± 0.04
Intermediate	18	0.11 ± 0.02
Poor-risk	5	0.16 ± 0.06
*p*-Value	-	0.4224

Age and gender were analyzed using the student t test, and FAB subtypes and karyotype subgroups were analyzed using one-way ANOVA test. The karyotype of 30/67 ND AML patients could be identified. ANOVA, analysis of variance.

**Table 4:** Primers used for qRT-PCR in human samples.

Gene	Primer Sequence (5′–3′)
hsa_circ_0035381-F	GGTGACTGTGCTGTGGAC
hsa_circ_0035381-R	TGGTGTTTGTAAGGGTTC
hsa_circ_0004136-F	ATGGCAAGGAAGACTGAG
hsa_circ_0004136-R	AGGGATGGTAGAAAACAC
hsa_circ_0058058-F	GGTGGTGTCCACGGAGAT
hsa_circ_0058058-R	CCAAGAGCGGTCAGGTTT
hsa_circ_0017446-F	GGAGCATAGAGACAGGGA
hsa_circ_0017446-R	GGCTTTTGTTTTGAGTTG
hsa_circ_0004277-F	CACTTACAAGGCTTCCAC
hsa_circ_0004277-R	CTTACTCAGCTCTGCTCC
WDR37-F	TTCCACCAGCAAGATTGTCTC
WDR37-R	GCGTACTTGACTAGGCACTTCC
GAPDH-F	GGGAAACTGTGGCGTGAT
GAPDH-R	GAGTGGGTGTCGCTGTTGA

## Abbreviations

circRNA	Circular RNA
AML	Acute myeloid leukemia
CN-AML	Cytogenetically normal AML
lncRNA	Long noncoding RNA
cRNA	Complementary RNA
ceRNA	Competing endogenous RNA
GO	Gene oncology
KEGG	Kyoto Encyclopedia of Genes and Genomes
SEM	Standard error of the mean
ND	Newly diagnosed
CR	Complete remission
RE	Relapsed-refractory
ROC	Receiver-operating characteristic
AUC	Area under the ROC curve
SRY	Sex-determining region Y
BM	Bone marrow
FLT3-ITD	FMS-like tyrosine kinase-3
NPM1	Nucleophosmin 1
qRT-PCR	quantitative Real-Time PCR
GAPDH	Glyceraldehyde 3-phosphate dehydrogenase
GEO	Gene Expression Omnibus
